# Challenge and opportunity for vector control strategies on key mosquito-borne diseases during the COVID-19 pandemic

**DOI:** 10.3389/fpubh.2023.1207293

**Published:** 2023-07-24

**Authors:** Hong-Zheng Lu, Yuan Sui, Neil F. Lobo, Florence Fouque, Chen Gao, Shenning Lu, Shan Lv, Sheng-Qun Deng, Duo-Quan Wang

**Affiliations:** ^1^Chinese Center for Disease Control and Prevention, National Institute of Parasitic Diseases, Shanghai, China; ^2^Department of Pathogen Biology, the Key Laboratory of Microbiology and Parasitology of Anhui Province, the Key Laboratory of Zoonoses of High Institutions in Anhui, School of Basic Medical Sciences, Anhui Medical University, Hefei, China; ^3^Department of Epidemiology and Biostatistics, School of Public Health, Anhui Medical University, Hefei, Anhui, China; ^4^Brown School, Washington University, St. Louis, MO, United States; ^5^Malaria Elimination Initiative, Institute for Global Health Sciences, University of California, San Francisco, San Francisco, CA, United States; ^6^Eck Institute for Global Health, University of Notre Dame, Notre Dame, IN, United States; ^7^Research for Implementation Unit, The Special Programme for Research and Training in Tropical Diseases, World Health Organization, Geneva, Switzerland; ^8^Chinese Center for Tropical Diseases Research, Shanghai, China; ^9^WHO Collaborating Centre for Tropical Diseases, Shanghai, China; ^10^National Center for International Research on Tropical Diseases, Ministry of Science and Technology, Shanghai, China; ^11^Key Laboratory of Parasite and Vector Biology, Ministry of Health, Shanghai, China; ^12^School of Global Health, Chinese Center for Tropical Diseases Research, Shanghai Jiao Tong University School of Medicine, Shanghai, China

**Keywords:** COVID-19, mosquito-borne diseases, vector control, mosquito biological control, mosquito novel control

## Abstract

Mosquito-borne diseases are major global health problems that threaten nearly half of the world’s population. Conflicting resources and infrastructure required by the coronavirus disease 2019 (COVID-19) global pandemic have resulted in the vector control process being more demanding than ever. Although novel vector control paradigms may have been more applicable and efficacious in these challenging settings, there were virtually no reports of novel strategies being developed or implemented during COVID-19 pandemic. Evidence shows that the COVID-19 pandemic has dramatically impacted the implementation of conventional mosquito vector measures. Varying degrees of disruptions in malaria control and insecticide-treated nets (ITNs) and indoor residual spray (IRS) distributions worldwide from 2020 to 2021 were reported. Control measures such as mosquito net distribution and community education were significantly reduced in sub-Saharan countries. The COVID-19 pandemic has provided an opportunity for innovative vector control technologies currently being developed. Releasing sterile or lethal gene-carrying male mosquitoes and novel biopesticides may have advantages that are not matched by traditional vector measures in the current context. Here, we review the effects of COVID-19 pandemic on current vector control measures from 2020 to 2021 and discuss the future direction of vector control, taking into account probable evolving conditions of the COVID-19 pandemic.

## Introduction

Mosquitoes are the most important vectors for disease transmission in terms of morbidity and mortality rates. Malaria, dengue fever, and yellow fever transmitted by mosquitoes have significantly high incidences, posing several public health problems (1). More than 600,000 people died of malaria in 2021 (2). Fifty to one hundred million people were infected with dengue fever annually, which leads to half a million hospitalizations (3). Infections by or continuous transmission of yellow fever, chikungunya, and Zika viruses also have significant public health impact, threatening more than 40% of the global population (4). Since the coronavirus disease 2019 (COVID-19) pandemic some low-income tropical or endemic countries have been unable to sustain funding for mosquito-borne diseases to ensure control (5).

Investment in the knowledge of the pathogenesis of these mosquito-borne diseases and antiviral drugs has increased exponentially over the past 20 years, but progress in the development of effective treatments, with the exception of malaria, remains slow (6). In the same way, the development of vaccines against mosquito-borne diseases has never reached its goals, with the exception of yellow fever. Therefore, for many vector-borne diseases, vector control remains the primary intervention to control mosquito-borne diseases through several techniques classified by physical, biological, chemical, genetic, and environmental aspects. Before the discovery of insecticides in the 1930s and the large-scale use of insecticides and mosquito nets, vector control interventions relied primarily on environmental management (7). The focus is on removing mosquito breeding sites and improving housing by installing screens to prevent mosquitoes from entering through doors and windows. This involves the installation of tight-fitting screened doors, screening or closing eaves, and replacing thatched roofs with solid materials such as metal or tile (8). In the past century, the deployment of insecticide-treated nets (ITNs), long-lasting insecticidal nets (LLINs), and indoor residual spraying (IRS) have become the primary and recommended means of mosquito vector control (7). Significant progress in malaria, dengue fever, and Zika viruses, the most important mosquito-borne diseases, has been achieved through the distribution of treated mosquito nets to at-risk populations and insecticide spraying. However, due to the cost issues, and operational constraints, traditional vector control measures are losing efficiency in controlling mosquito-borne diseases. The benefits from these techniques are gradually plateauing (9). Although multiple strategies are being used and the development of LLINs and IRS with different compounds is accelerating, the global burden of mosquito-borne diseases on public health and economies continues to increase (10).

On March 11, 2020, the World Health Organization (WHO) declared COVID-19 a global pandemic (11). Confirmed cases emerged in more than 200 countries. Along with the long-standing challenges of globalization, climate change, urbanization and insecticide resistance, the mosquito vector control process within the COVID-19 context was facing unprecedented difficulties, further highlighting the need for new technologies and strategies. A series of challenges will prevent the critical goals of the WHO Global Strategy on malaria for 2030 from being met (12). Therefore, we must change our thinking and adopt innovative and transformative approaches to vector control. Biological control, represented by the release of sterile male mosquitoes and new biocides, has excellent potential, but no reports of novel strategies were developed or implemented during COVID-19. In this review, we explore the interaction between COVID-19 and mosquito-borne diseases, and we discuss promising vector control strategies within the current environment. Data for this review were initially identified through a search of PubMed, Web of Science and ScienceDirect. We independently extracted and recorded data from each eligible study. Specific content and reasons for the implementation of mosquito vector measures affecting COVID-19 were manually screened to ensure accuracy. We ended up including 19 articles in Table 1 that describe the impacts from 2020 to 2021.

**Table 1:** The effects of COVID-19 on traditional vector control strategies.

Region	Location	Time	Influence	Reason	Refs
Africa	Burundi	2020 to 2021	Severe shortage of health personnel, lack of conventional vector control products	COVID-19 affects the economic level leading to inflationary problems	(13)
	Congo	2020	LLIN mass distribution campaign covering approximately 59 million people in 14 provinces suspended	COVID-19 pandemic outbreak dominated the political and health agenda in March 2020	(14)
	Comoros	2020	Postponement of ITN and IRS activities	Health system overwhelmed	(15)
	Côte d’Ivoire	2020	Postponement of ITN and IRS activities	Health system overwhelmed	(16)
	Eswatini	2020	COVID-19 pandemic slow and complicate the planning and preparation of malaria control programs (e.g., IRS)	The COVID-19 pandemic limits the movement of people	(17)
	Southern Mozambique	2020	COVID-19 pandemic slow and complicate the planning and preparation of malaria control programs (e.g., IRS)	The COVID-19 pandemic limits the movement of people	(17)
	Northern Ghana	January to April 2020	Closure of malaria clinics, cessation of routine ITN distribution	Lockdown	(18)
	Kenya	2021	Median monthly LLIN distribution declines, mass community distribution campaign delayed by 10 Months	COVID-19 lockdown strategy and health workers strike	(13)
	Zimbabwe	2020	IRS and ITN distribution suspended, malaria commodity shortage	Lockdown, curfews, access restrictions due to the COVID-19 pandemic	(19)
America	Brazil	2020	Dengue budget cuts (including the budget for hospitalization and vector control measures, such as ITN and IRS)	Health system announced priority for surveillance and virus identification of COVID-19	(20, 21)
	Coastal Ecuador	2020	Suspension of routine mosquito vector monitoring and control programs	Coinfection of dengue fever and SARS-CoV-2 viruses	(22–24)
	Honduras	2020	Suspension of routine mosquito vector monitoring and control programs	Two hurricanes and a sharp increase in COVID-19 cases	(24, 25)
Asia					
	Afghanistan	2021	Inefficient mosquito net distribution services and implementation of the Sehatmandi project were hampered	Internal Conflict and COVID-19 pandemic	(26)
	Bhutan	March to May 2020	Mass distribution of LLIN program delayed, IRS, health education, and mosquito vector surveillance disrupted	COVID-19 pandemic caused freight disruptions, affecting movement of goods and people	(27)
	Meghalaya State, India	2020	National Vector Borne Disease Control Program conducted in 50 villages, 7 of which were unable to conduct IRS activities	Movement restrictions due to COVID-19	(28)
	Pakistan	2020	Water disinfection plan put on hold and dengue outbreak	Flooding Outbreak and Funds Used for COVID-19 Prevention Program	(29)
Europe	France	2020	Vector control interventions in all overseas sectors were reduced, social mobilization campaigns were put on hold, and preventive insecticide spraying in private premises was curtailed	Lockdown	(30, 31)

## Effects of COVID-19 on current mosquito vector control measures

In the early days of the COVID-19 pandemic, most countries took measures in the form of lockdowns. In the short term, the lockdown may have had a positive impact on mosquito-borne diseases by preventing regional or inter-country transmission of infected individuals (32). In parallel, the vector will continue to reproduce and host-seek on humans, alongside a reduction in access to health facilities and health professionals combined with supply chain issues. As more countries opened their borders and stopped the lockdown, the need to reconsider how they balanced COVID-19 with other epidemics appeared. For example, the complete closure of health and vector control team activity during the lockdown may have resulted in increased vector populations (33). Common mosquito-borne diseases, such as Zika, dengue fever, and malaria, are at risk of outbreaks (34, 35). Additionally, the co-infection of SARS-CoV-2 and dengue fever viruses have imposed a significant burden on healthcare systems in dengue-endemic regions (36). India imposed its first nationwide lockdown on March 24, 2020. Observations were conducted in two areas of Bangalore, India, comparing data before and after the lockdown (February to April 2020, collected once a month). Compared to February, the *Aedes aegypti* house index and Breteau index increased from 6.6 and 9.3 to 26.6 and 34.6, respectively. Very significant increase compared to 2017 to 2019 data for this location (37). In addition to India, mosquito larval site monitoring in Sri Lanka, Cuba, Indonesia, and Malaysia demonstrated varying increases (38–41). This suggests that the probability of mosquito-borne disease outbreaks may increase in places where mosquito populations become larger. Within this context, mosquito vector control measures should not be reduced or abandoned but should be given more attention. Furthermore, lockdown measures obliged people to stay much more in their home, where the transmission of arboviral diseases usually occurs. Cavany et al. (42) used a model to predict changes in dengue incidence due to lockdown, with the proportion of people infected in their own homes increasing from 54% in normal conditions to 66% in lockdown conditions, and the rate of secondary household attacks increasing from 0.109 to 0.128, a 17% increase.

The immediate effect of COVID-19 on mosquito vector control measures was the massive diversion of medical resources. Budgets for actions, such as IRS and ITNs, have been massively cut (43). ITN and IRS defined by WHO as cornerstones of mosquito vector control. The rapid delivery of ITNs to populations at risk of mosquito-borne diseases in a remarkably short period of time through mass campaigns is currently the primary method of ITN operation (44). Governments, private sectors, and religious and humanitarian organizations have been working on this for the past few decades, and much has been accomplished. Sleeping under ITNs has reduced the incidence rate of malaria by 50% (45). Since the declaration of COVID-19 as a pandemic, attention has shifted to COVID-19, interrupting several intervention programs for equally health-threatening infectious diseases (46). Simultaneously, control activities, such as the distribution of mosquito nets and community education, ceased or were significantly reduced (43). The highest levels of mosquito-borne diseases have been found in sub-Saharan Africa for the past 20 years, and those regions were then the ones suffering most of the consequences of COVID-19 disruptions. Furthermore, after the COVID-19 pandemic, the crowding out of medical resources, diversion of funds, and interruption of logistics caused by the embargo made the original control measures impossible to implement (47). Overall, the impacts of the COVID-19 pandemic during 2020 and 2021 were much larger than envisioned for several mosquito-borne diseases on different continents (Table 1).

Varying degrees of disruptions in malaria control and ITNs and IRS distribution worldwide from 2020 to 2021 were reported (Table 1). According to the WHO, less than half of the 22 million ITNs planned for global distribution in 2020 had been distributed as of November 2020. Meanwhile, less than half of the routine IRS activities in malaria-endemic countries have been completed (48). A majority (58%) of countries (out of 64) report disruptions in the service delivery of their malaria control programs from 2020 to 2021 (5). Reducing IRS and ITN allocation is expected to lead to severe consequences. In the most extreme scenario, conventional malaria control measures, including a 75% reduction in ITN distribution and drug shortages, would increase sub-Saharan malaria morbidity and mortality rates by more than 20 and 50%, respectively (49). Hogan et al. (50) predicted the extent of disruption to healthcare and malaria control services during the COVID-19 pandemic. They estimated that the global malaria mortality rate could increase by 36% over the next 5 years, mainly due to a shortage of ITNs and the scarcity of other essential commodities. In addition, ITNs and IRS are labor-intensive vector control measures, the implementation of which inevitably leads to interaction between communities, in contradiction with the recommendation on COVID-19 (to avoid crowding). For this reason, the budget for COVID-19 personal protective equipment in several activities has been increased (51). Creating more outdoor facilities and improving indoor air circulation in residential and commercial buildings to reduce the risk of COVID-19 may increase exposure to mosquitoes (52).

In addition to the consequences of the COVID-19 pandemic, several challenges are currently faced by conventional vector control activities, such as the costs of implementation, traditional mosquito vector measures, slow operational implementation, and insecticide resistance (53). The increasing trend of insecticide resistance observed in recent years is alarming, and resistance to four classes of insecticides (pyrethroids, organochlorines, carbamates, and organophosphates) was reported in 32% of the countries with mosquito-borne disease transmission. The newest approved ingredient in IRS products, clothianidin, has already been resistant in Central Africa (54). Moreover, 90% of malaria-endemic countries have reported resistance to at least one class of insecticides in *Anopheles* (9). Although ITN- and IRS-led vector control methods remain valid today against malaria, their lifespan is shortened. When the COVID-19 outbreak became a pandemic, new, transformative, and innovative vector control technologies were already required and are now more strongly necessary to address the current situation.

### Innovative vector control strategies in development in the current context

Since early 2020, some countries have combined modern technology with traditional vector interventions to facilitate follow-up. For example, the ITN distribution was monitored through digital technology with a mobile application for timely monitoring and supervisory feedback, allowing more rapid collection of household statistics and ITN distribution data. This technology allowed for avoiding contact with personnel to a certain extent (51, 55).

A prior Mexican study used unmanned aerial vehicles (UAVs) to identify *Ae. aegypti* breeding sites and spraying to reduce the need for field technicians, achieving a 64.9% agreement between UAVs and ground monitoring. Moreover, UAVs can access breeding sites that cannot be accessed or identified by traditional ground monitoring and disinfection and ensure that routine disinfection and monitoring is conducted during the lockdown (56). In another case, Gabriel Carrasco-Escobar et al. used drones in Peru to identify *Anopheles darlingi* breeding sites through high-resolution images and multispectral profiles with an overall accuracy of 86.73–96.98% (57). In addition to collecting mosquito habitats and disinfecting them, drones are valuable tools for monitoring the environmental factors that influence disease dynamics. Flaviviruses are primarily maintained by wild, non-human primate hosts, and drones can map the migration patterns of wildlife populations and changes in their habitats. This brings benefits for real-time monitoring of disease dynamics, as well as vector intervention programs (58, 59).

### Releasing sterile or lethal gene-carrying male mosquitoes

Releasing male mosquitoes as biological insecticides is a cutting-edge technology with great promise. These technologies are based on gram-negative intracytoplasmic bacteria of the genus *Wolbachia*, found in 76% of the world’s insect species and is the most widely distributed commensal bacterium worldwide (60). Manipulation of *Wolbachia* strains can induce anti-RNA viral properties in its hosts, inhibiting the development of pathogens, such as dengue virus and chikungunya virus, in mosquito vectors (61, 62) and is also associated with several reproductive operations in mosquito vectors (63). The result of the CI will be the suppression of the mosquito population. Therefore, CI-based population control is referred to as an incompatible insect technique (IIT). Different from conventional mosquito vector control methods, IIT involves the regular release of *Wolbachia*-carrying male mosquito populations with appropriate methods to reduce the mosquito population size, thus achieving the goal of disease control. With the study of the principle of CI and the development of embryo microinjection techniques, progress has also been made in important mosquito vectors that do not naturally carry *Wolbachia* by injecting infected insect cytoplasm or tissue into mosquito embryos (64). Several successful trials have shown positive results of *Wolbachia* in mosquito vector control (65–67).

The sterile insect technique releases large numbers of sterile male mosquitoes to mate with wild females (68). Sterility methods include chemical, radiation, hybrid sterility, and chromosomal translocation, of which radiation sterility is the most commonly used. SIT has the advantages of being environmentally friendly and controllable on a large factory scale. For decades, SIT has achieved many successes in agricultural control and population suppression, and it is currently widely tested against *Culex*, *Anopheles* and *Aedes* mosquitoes (69–71). An example, among many others, of an SIT field trial, was conducted during the COVID-19 pandemic in southern Germany infested with *Ae. albopictus*. Continued release of sterile male mosquitoes from May to September 2020 was achieved in the trial areas of Ludwigshafen and Freiburg, with egg sterility reaching 84.7 ± 12.5% and 62.7 ± 25.8%, respectively; in comparison, the natural sterility in the control area was 14.6 ± 7.3% (72).

Genetic sterility was also used with the release of insects with a dominant lethality gene (RIDL). The corresponding gene expression in the target population is introduced by releasing male transgenic mosquitoes carrying the dominant lethal gene. The expression of dominant lethal genes in the currently developed RIDL system is regulated by the Tet-Off system. The lethal gene is under the control of the tetracycline resistance operon (tetO), a response element of the tetracycline transcriptional activator (tTA). In the absence of tetracycline, the tTA activator binds tetO and activates the promoter to induce the expression of dominant lethal genes. In contrast, in the presence of tetracycline, tTA binds to tetracycline and prevents it from binding to the tetO site, thereby inhibiting the system (73). In the wild, the offspring of RIDL mosquitoes express the gene because of the lack of tetracycline in their diet, thus achieving control of population density. Compared to SIT, RIDL does not require manual separation of males and females, the sex-specific promoter separates males from females, and there is no reduction in the competitive ability of males (74). Various RIDL strains have been developed, including *A. aegypti*, *A. albopictus*, and *Anopheles gambiae*, which are conditionally lethal, specifically lethal, and wingless (73, 75–77).

Extensive trials have demonstrated the feasibility and unique advantages of releasing sterile or lethal gene-carrying male mosquitoes for mosquito vector control. In addition to the absence of insecticide resistance problems associated with traditional methods, long-term cost-saving benefits will address the current funding shortfall due to COVID-19 (78). In terms of implementation and effectiveness, there are advantages to using new technologies for mosquito control that are difficult to match with traditional methods in the current environment. The biggest challenge for the release of male mosquitoes carrying sterile or lethal genes is transportation. It is crucial that they arrive at the release site within 24 h; otherwise, their survival rate, flight ability, and mating ability can be negatively affected (79). Unfortunately, the absence of a globally common procedure for the transport of male mosquitoes makes it challenging to use these new technologies on a large scale in developing countries. However, combining multiple control tools and methods, such as geographic information systems, spatial analysis, or UAV could potentially improve the current situation and increase sustainability (80, 81). Other than that, most of these innovative technologies also have drawbacks that are not yet fully overcome. Larval rearing, field monitoring, selection of suitable strains, and construction of models with optimal solutions for release frequency and time to achieve the best release strategy have essential effects on control effectiveness (82). To optimize the utilization of resources and ensure the sustainability of the control program, new technologies must be integrated with a risk stratification system. Incorporating efficient predictive models and a centralized monitoring approach will significantly enhance the practicality of adopting these new technologies (83).

### Novel biopesticides

In addition, biopesticides have become popular in recent years and have certain advantages in the current context. Fungi and bacteria are the main focus of current biopesticide research. The mechanism of action between the fungus and host is significantly complex and divided into several stages of adhesion, penetration, and colonization. Fungal spores invade the epidermis and break open the body wall by forming infestation structures, interfering with the metabolic function of the host and secreting toxins (84–86). From the perspective of mosquito control mechanisms, the fungus is highly suitable for on-site mosquito control. The fungus can attach to mosquito carcasses to reproduce and create an epidemic within the mosquitoes for continuous power. Mosquitoes with fungal disease can carry fungal spores to other mosquito habitats to infect more mosquitoes (87). Fungal biopesticides have low developmental costs, are convenient to use, and have considerable effects. There are no reports on mosquito resistance to fungal biopesticides (88). These properties make fungal insecticides promising for mosquito control. *Metarhizium anisopliae* and *Beauveria bassiana* are more developed than other fungi in fungal mosquito control. They can shorten the lifespan of many mosquitoes, including *Anopheles*, *Aedes*, and *Culex* (89–91). Mosquitoes exposed to *M. anisopliae* and *B. bassiana* die within 3–14 days, and their desire to suck blood and reproduce is reduced (87, 92). In addition to affecting survival time and reproductive capacity, *M. anisopliae* and *B. bassiana* have inhibitory effects on mosquito pathogens. Fang found that recombinant *M. anisopliae* can prevent the development of *Plasmodium* in the vector and can reduce the number of sporozoites by 98%, indicating that *M. anisopliae* is effective in fighting against malaria (93). Deng found that Zika virus (ZIKV) titer levels in the midgut, head and salivary glands were significantly reduced after feeding ZIKV to *Ae. albopictus* females (94). Another study found that *Ae. aegypti* infected with both *M. anisopliae* and dengue virus type 2 (DENV-2), and the infection rates of DENV-2 in the heads and midguts were significantly reduced (95). As abiotic factors (temperature, humidity, and ultraviolet radiation) affect the effectiveness of fungi in field applications, and the low virulence of the fungus leads to low efficiency of mosquito killing has been an important reason for its popularity is not widespread. Therefore some researchers have inserted some natural and synthetic genes into the fungal genome to improve their virulence and tolerance (96). For example, heat-tolerant genes can be genetically engineered into *M. anisopliae* to enhance their adaptability (97). *Androctonus australis* insect toxin is a neurotoxin widely used for recombinant expression in fungi (98). A prior study reported the toxicity of the recombinant *M. anisopliae* formed by this gene in adult *Ae. aegypti* increased by nine times (99). In addition, many other genes, such as [SM1]_8_ and scorpine, were recombined into pathogenic fungi to enhance their control of mosquito-borne infectious diseases (93).

The most mature bacterial biopesticide is *Bacillus thuringiensis*. It is a gram-positive, rod-shaped, spore-forming bacterium with facultative oxygen demand. Its toxin is mainly present in accompanying spore crystals formed during the development of budding spores (100). *B. thuringiensis* is active against Lepidoptera, Coleoptera, Diptera, Hymenoptera, Homoptera, Orthoptera, and Nematoda, but it is not toxic to mammals (101, 102). The development of *B. thuringiensis* var. *israelensis* enables *B. thuringiensis* to be invested in mosquito control on a large scale. *B. thuringiensis var. israelensis* is effective against 72 species of mosquitoes (21 species of *Anopheles*, 21 species of *Aedes*, and 17 species of *Culex*) (103). As a biofriendly insecticide with high mosquito control efficiency, convenient storage, and application forms, *B. thuringiensis* has been used in many applications worldwide. The primary tool for mosquito control in Hawaii, United States, is *B. thuringiensis* (104, 105). *B. thuringiensis* in drinking water is harmless to humans and well suited for use as a household-level biocide, and its resistance is difficult to pass on to mosquitoes (106). These characteristics make the number of *B. thuringiensis* unlimited in the field of mosquito control, providing a powerful alternative for mosquito control in the current environment. Another bacterium that has been relatively successful in mosquito killing is *Bacillus sphericus*. *B. thuringiensis* and *B. sphericus* have their own advantages and disadvantages; *B. thuringiensis* has a broad spectrum of insecticides, while *B. sphericus* has a long shelf life (107).

Biological insecticides are more widely used in practical applications than the release of male mosquitoes for control. They are currently used mainly in combination with traditional chemical insecticides to delay the problem of drug resistance. As an environmentally friendly tool that is also highly specific and can limit the growth of target populations in successive generations after application, its advantages are clear. It is highly suitable for promotion in the current environment to address resistance and cost issues (108). Biological control, represented by the release of male mosquitoes and biopesticides, can solve many of the pain points of traditional vector measures in the current context (Table 2).

**Table 2:** Comparison of traditional vector control (ITNs/LLINs/IRS) and new vector control technologies (the release of male Mosquitoes and biopesticides).

	ITNs/LLINs/IRS	The release of male mosquitoes	Biopesticides	Refs
Drug resistance	Presence	Not applicable	Complex mechanisms make it difficult to pass on drug resistance	(9, 88, 106, 109)
Application method	Person-to-person contact	Low labor intensity, targeting mosquitoes, no human contact	Person-to-person contact	(52, 78)
Cost	The efficiency of mosquito control decreases every year with the increase in drug resistance, and the cost also increases	saving health personnel, long-term cost-saving benefits	Wide range of sources, cost-effective, easy to raise and use	(78, 110, 111)
Sustainability	Needs to be applied or replaced regularly	Sustainable control	can limit the growth of target populations in successive generations after the application	(108, 112, 113)
Environmental impact	negative impact	Unknown	Environmentally friendly	(108, 114)
Effects on the human body	negative impact	No negative impact	High specificity and no negative impact	(108, 114, 115)

In recent years, natural repellents have gained increasing attention due to their pure plant ingredients which are low in residue and easy to degrade. They are also known for being low or non-toxic, having minimal skin irritation effects and being environmentally friendly (116). Natural repellents are primarily derived from various plant parts such as stems, roots, leaves, flowers, and fruits, among others. The active ingredients of these repellents are mostly esters, ketones, and alcohols of terpenoids, and they often contain flavonoids and alkaloids, among others. Currently, the focus on the development of natural repellents involves the extraction of natural plant products and the analysis of their active ingredients. This analysis is considered a hot issue in natural repellent research (117). In addition to the technologies described above, several mosquito vector control technologies are still being developed, including acoustic larvicides, RNAi-based biocides, and nanotechnology, which are equally desirable. In general, the situation of mosquito-borne diseases during the COVID-19 pandemic is serious, and the application and promotion of new vector control strategies should be strengthened to effectively reduce the spread of mosquito-borne infectious diseases.

## Conclusion

Although the new mosquito vector control technologies introduced above have significant advantages over traditional strategies, the market and practical applications are still dominated by traditional methods. There are several reasons for this phenomenon.

- The market for new technologies is difficult to guarantee, resulting in insufficient motivation and funding for research and development, forming a vicious circle.
- Alternative and novel ways of approaching the issue may combat historical and habitual thinking, allowing new paradigms to combat the current transmission.
- Previous mosquito vector control focused more on quick results, especially chemical insecticide-led vector control, which ignored the long-term benefits and environmental and ecological effects. A scale-down of these control measures invariably leads to an immediate increase in vectors.
- The standardization process is slow, and larger-scale field trials cannot be conducted based on the funding needed.

The COVID-19 pandemic has exposed weaknesses in some countries’ preparedness and response capacity for public health crises and the inadequacy of existing mosquito vector control systems. The COVID-19 pandemic has had a significant effect on mosquito vector control and, in its aftermath, brings a huge opportunity to improve old strategies or develop more efficient and resilient ones. At the national level, epidemics have received unprecedented attention and some tilt of resources to public health. Systems and structures established as a result of pandemics may also present new opportunities for mosquito-borne disease control; for example, the strengthening of community infrastructure and the system established by the state for monitoring the spread of COVID-19 also facilitate mosquito-borne disease projects. Simultaneously, laboratory capacity has improved in many countries, and the increased power of sequencing technology and increased level of testing can also be used to strengthen mosquito vector surveillance activities. At the individual level, more people are willing to learn about this aspect and pay more attention to epidemic prevention in their daily lives, which will be helpful for future health education campaigns on mosquito-borne diseases and the promotion and implementation of mosquito-borne measures. To alleviate the current dilemma of mosquito-borne disease in the context of the COVID-19 and to prevent mosquito-borne disease from becoming the successive COVID-19, a change in concept, the development of new technology research, and the accelerated operation of field trials are needed.

## Author contributions

All authors listed have made a substantial, direct, and intellectual contribution to the work and approved it for publication.

## Funding

This paper was funded by China–Africa cooperation project on malaria control under the Project (No. 2020-C4-0002-3), the program of the Chinese Center for Tropical Diseases Research (no.131031104000160004) as well as UNICEF/UNDP/World Bank/WHO Special Program for Research and Training in Tropical Diseases (TDR) Small Grant (WHO Reference 2021/1104003–0) to WDQ, and National Natural Science Foundation of China (NO. 8210082025) and Anhui Provincial Natural Science Foundation Project (no. 2108085QH347) to DSQ.

## Conflict of interest

The authors declare that the research was conducted in the absence of any commercial or financial relationships that could be construed as a potential conflict of interest.

## Publisher’s note

All claims expressed in this article are solely those of the authors and do not necessarily represent those of their affiliated organizations, or those of the publisher, the editors and the reviewers. Any product that may be evaluated in this article, or claim that may be made by its manufacturer, is not guaranteed or endorsed by the publisher.

## Abbreviations

CI: cytoplasmic incompatibility
COVID-19: coronavirus disease 2019
DENV-2: dengue virus type 2
IIT: incompatible insect technique
IRS: indoor residual spraying
ITNs: insecticide-treated nets
LLINs: long-lasting insecticidal nets
RIDL: the release of insects with a dominant lethality gene
SIT: sterile insect technique; tetO: tetracycline resistance operon
tTA: tetracycline transcriptional activator
UAVs: unmanned aerial vehicles
WHO: World Health Organization
ZIKV: Zika virus.
